# Supplementary material for: Polycomb complexes associate with enhancers and promote oncogenic transcriptional programs in cancer through multiple mechanisms
Source: Nat Commun. 2018 Aug 23;9:3377. doi: 10.1038/s41467-018-05728-x (PMC6107513; doi:10.1038/s41467-018-05728-x)
Supplement: Supplementary file 1 — Supplementary Information [file 41467_2018_5728_MOESM1_ESM.pdf]

Polycomb complexes associate with enhancers and promote oncogenic transcriptional programs in cancer through multiple mechanisms

Chan et al.

Supplementary Figures 1-10

Supplementary Figure 1

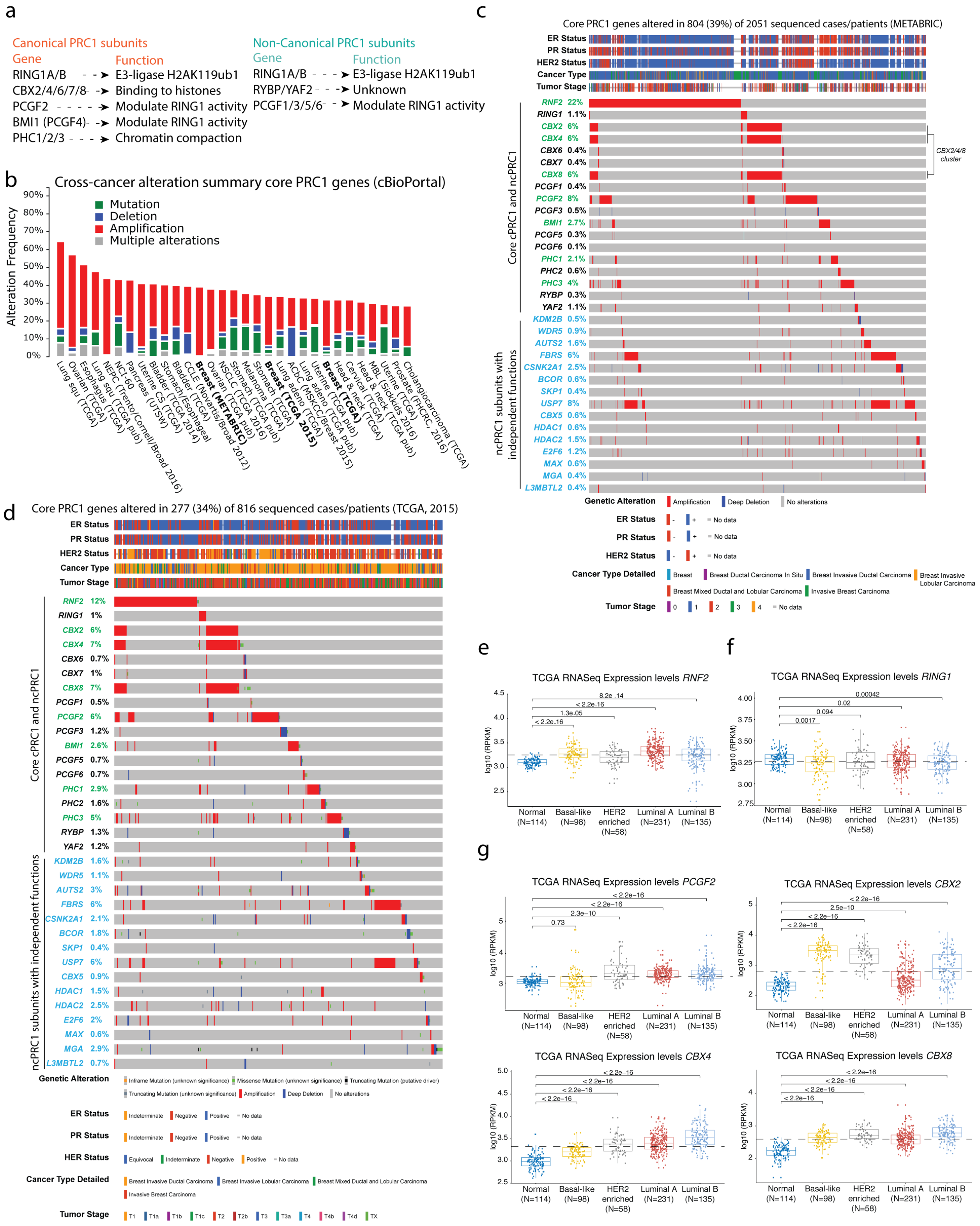

Supplementary Figure 1. Gain-of-Function and Amplification of Genes Encoding cPRC1 Components in Breast Cancer (**a**) List of canonical and non-canonical core PRC1 genes and their molecular function. (**b**) Genomic alteration status of genes encoding cPRC1 and ncPRC1 genes in different cancer types from published TCGA and the other datasets specified at the bottom. Alteration frequency refers to % of patients with at least one core PRC1 gene altered. (**c**) cPRC1 genes and *RNF2*, which encodes for RING1B, are amplified in breast cancer patient samples. Patient data is extracted from the METABRIC dataset. Expression of ERα, PR, and HER2 hormones are shown at the top. The *CBX2*/*4*/*8* genes are located within a genomic cluster. Genes highlighted in green are significantly amplified. Genes highlighted in blue are ncPRC1 genes that are part of other multiprotein complexes and have PRC1-independent function. (**d**) Amplification of core PRC1 and *RNF2* genes in the TCGA 2015 breast cancer dataset that includes 816 patient samples. Genes highlighted in green are the significantly amplified. Genes highlighted in blue are the ncPRC1 genes that are part of other multiprotein complexes and have PRC1-independent function. (**e–g**) *RNF2*, *RING1*, *PCGF*2, and *CBX2*/*4*/*8* mRNA expression analyzed from 1,211 breast cancer patients extracted from the TCGA breast cancer 2017 dataset. Gene expression is represented as $\log_{10}$ (RPKM). The number of patient samples in each molecular subtype is shown at the bottom. Center line of box plots represent the median and upper and lower bounds of whiskers represent the maximum and minimum values, respectively. Significance was determined using a non-parametric Wilcox test and p-values are indicated above each comparison.

Supplementary Figure 2

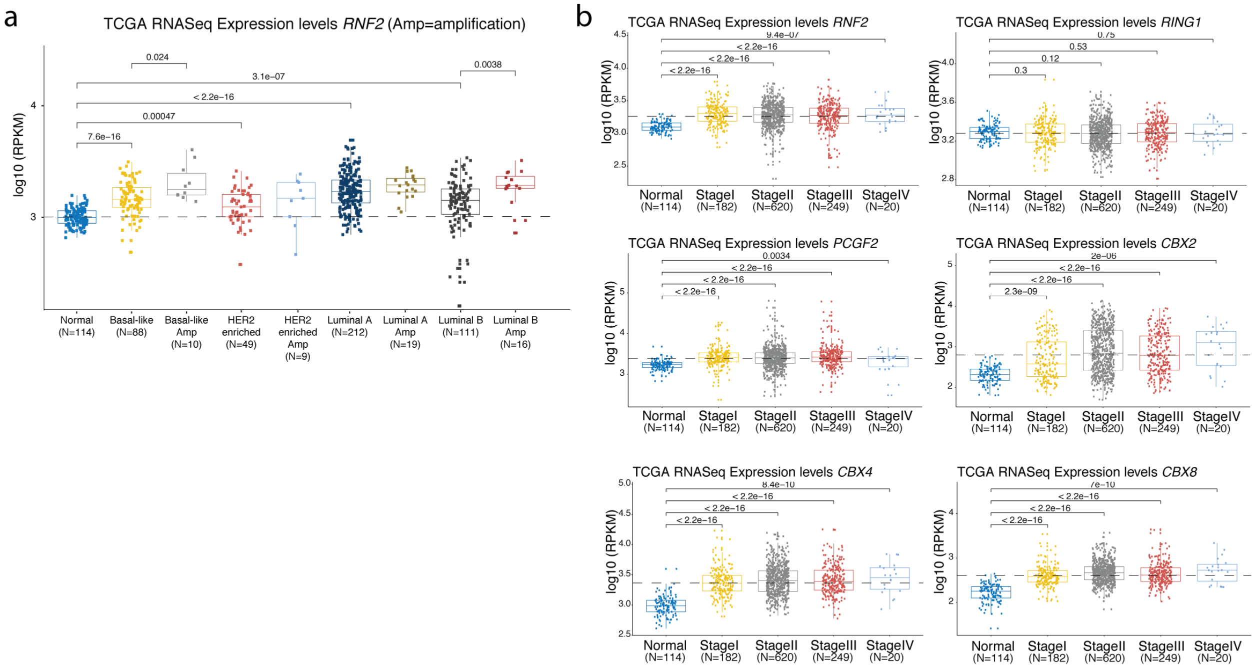

Supplementary Figure 2. Extended Analysis of PRC1-Encoding Gene Alterations in breast cancer. (**a**) *RNF2* expression was analyzed from patient samples with or without amplification (AMP) of *RNF2*, as indicated. The number of patients in each molecular subtype is shown at the bottom. (**b**) Expression of *RNF2*, *PCGF2*, *RING1*, and *CBX2/4/8* in the four breast cancer stages compared to normal breast tissues. Gene expression is represented as log10 (RPKM). The number of patients in each molecular subtype is shown. Center line of box plots represent the median and upper and lower bounds of whiskers represent the maximum and minimum values, respectively. Significance was determined using a non-parametric Wilcox test and p-values are indicated above each comparison.

Supplementary Figure 3

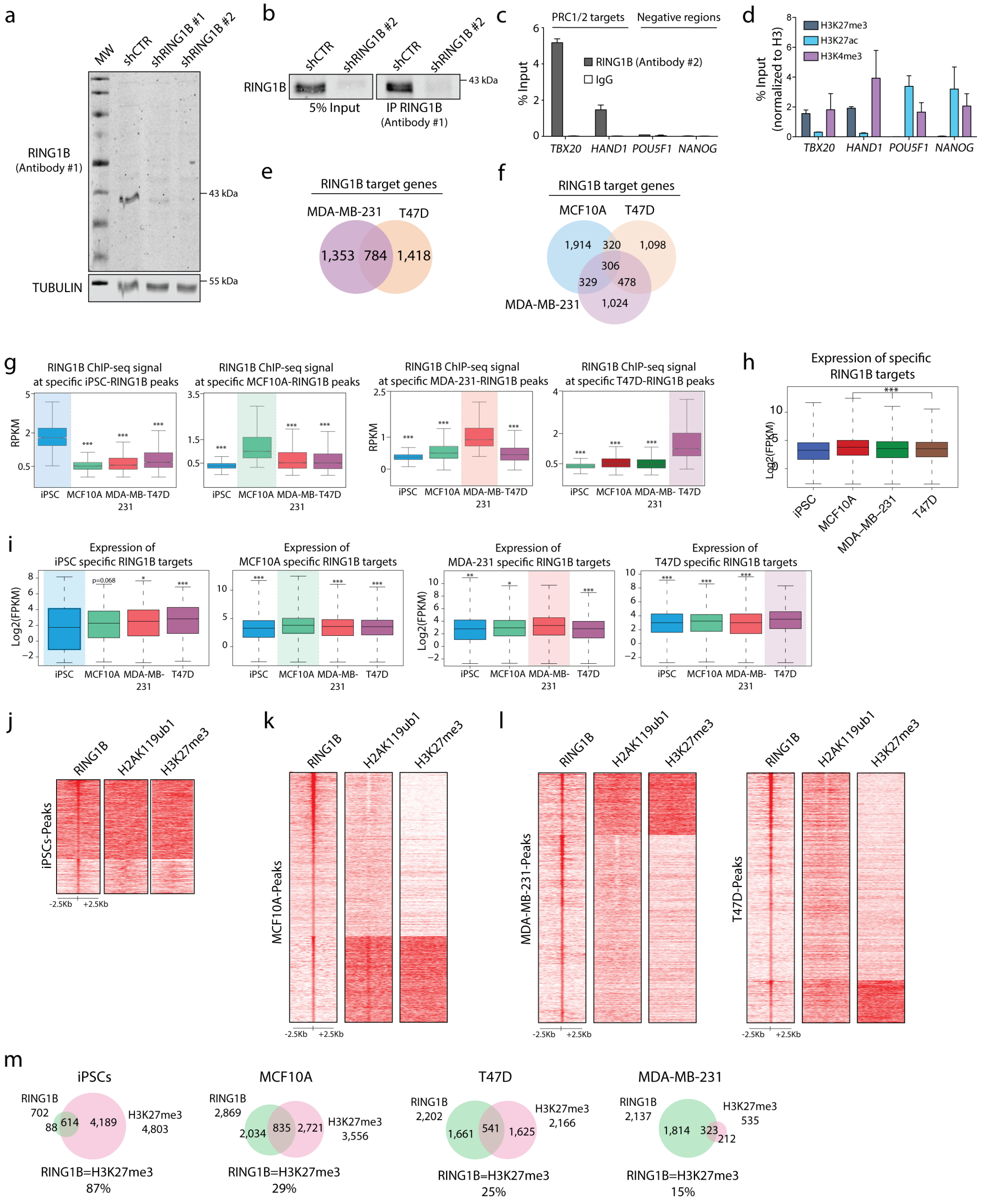

Supplementary Figure 3. Extended Characterization of RING1B target genes. (a) Uncropped western blot of whole-cell extracts from control and two shRNAs against RING1B in MDA-MB-231 cells. TUBULIN is used as a loading control. RING1B western-blot and ChIP-

seq were performed with an anti-RING1B antibody from Active Motif. (**b**) Endogenous RING1B IP in control and RING1B-depleted MDA-MB-231 cells. (**c**) RING1B ChIP-qPCR of PRC1/2 target genes (*TBX20* and *HAND1*) and pluripotency genes (*POU5F1* and *NANOG*) in iPSCs. IgG antibody was used as a negative control. Data is represented as percentage of input immunoprecipitated. RING1B ChIPs were performed with an anti-RING1B antibody from Cell Signaling. Error bars represent SD of two independent experiments (**d**) ChIP-qPCR of PRC1/2 target genes and pluripotency genes in iPSCs, performed with the antibodies indicated in the top. Results are presented as a percentage of input immunoprecipitated normalized by the histone H3 levels in each locus. Error bars represent SD of two independent experiments. (**e**) Venn diagram of RING1B target genes in MDA-MB-231 and T47D cells. (**f**) Venn diagram of RING1B target genes in MCF10A, MDA-MB-231 and T47D cells. (**g**) Box-plots of RING1B ChIP-seq signal (represented as RPKM values) in specific RING1B peaks detected in iPSCs, MCF10A, MDA-MB-231 and T47D cells. Significance was determined by the Mann-Whitney test. ***, p-value < 0.001. (**h**) Expression of cell type-specific RING1B target genes. RING1B target genes in MCF10A, T47D and MDA-MB-231 cells are significantly more expressed that RING1B targets in iPSCs. Significance was determined by the Mann-Whitney test. ***, p-value < 0.001. (**i**) Expression of the specific RING1B targets for each cell line (highlighted) compared with their expression in the other cell lines. Significance was determined by the Mann-Whitney test. *, p-value < 0.05; **, p-value < 0.01; ***, p-value < 0.001. (**j-l**) ChIP-seq heat maps of RING1B peaks containing H3K27me3 and H2AK119ub1 in MCF10A, MDA-MB-231 and T47D cells. (**m**) Venn diagrams of RING1B and H3K27me3 target genes in MCF10A, MDA-MB-231 and T47D cells.

Supplementary Figure 4

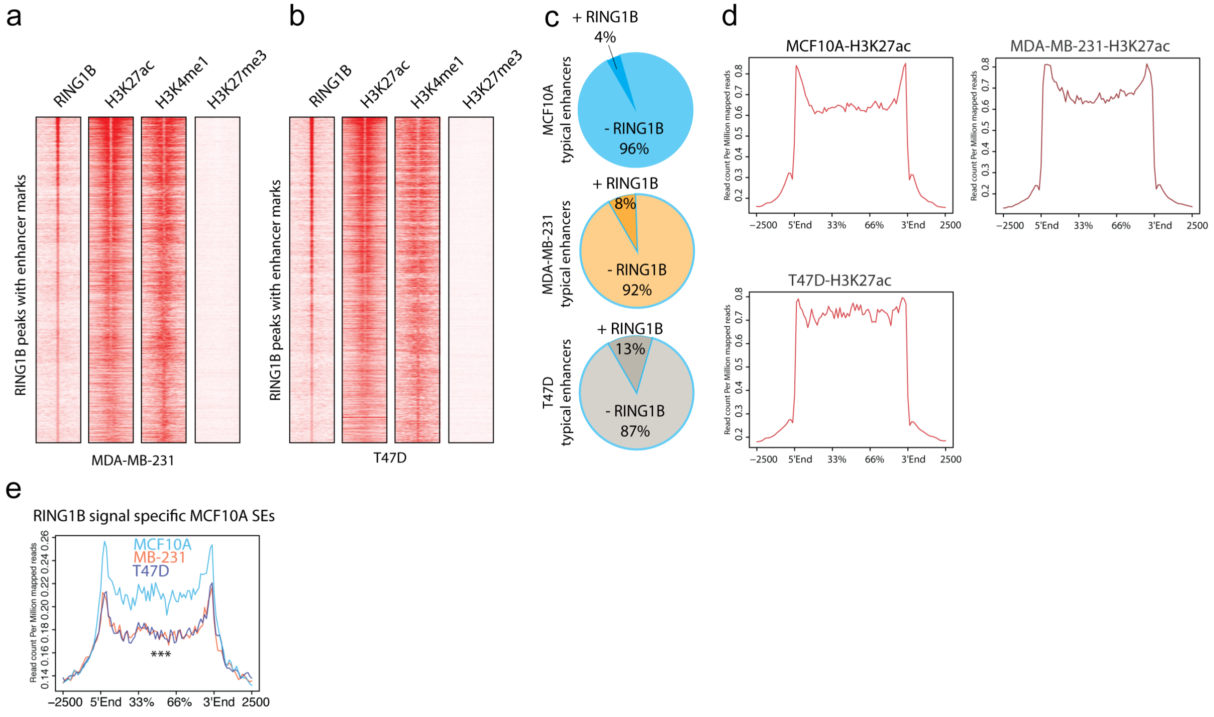

Supplementary Figure 4. Characterization of RING1B recruitment to enhancers. (**a, b**) ChIP-seq heat maps of RING1B peaks containing H3K27me3 and H3K4me1 in MDA-MB-231 and T47D cells. (**c**) Pie charts of typical enhancers with and without RING1B in MCF10A, T47D and MDA-MB-231 cells. (**d**) H3K27ac ChIP-seq profiles in MCF10A, T47D and MDA-MB-231 cells at the super-enhancers (SEs) identified in Figure 3B. (**e**) RING1B ChIP-seq signal of MCF10A, MDA-MB-231, and T47D at MCF10A specific SE regions. RING1B ChIP-seq signal in RING1B-MCF0A SEs compared to RING1B ChIP-seq signal in the same genomic region in T47D (p-value= 1.93e-12) and MDA-MB-231 (p-value= 2.65e-12). Significance was determined by the Kolmogorov–Smirnov test. ***, p-value < 0.001

Supplementary Figure 5

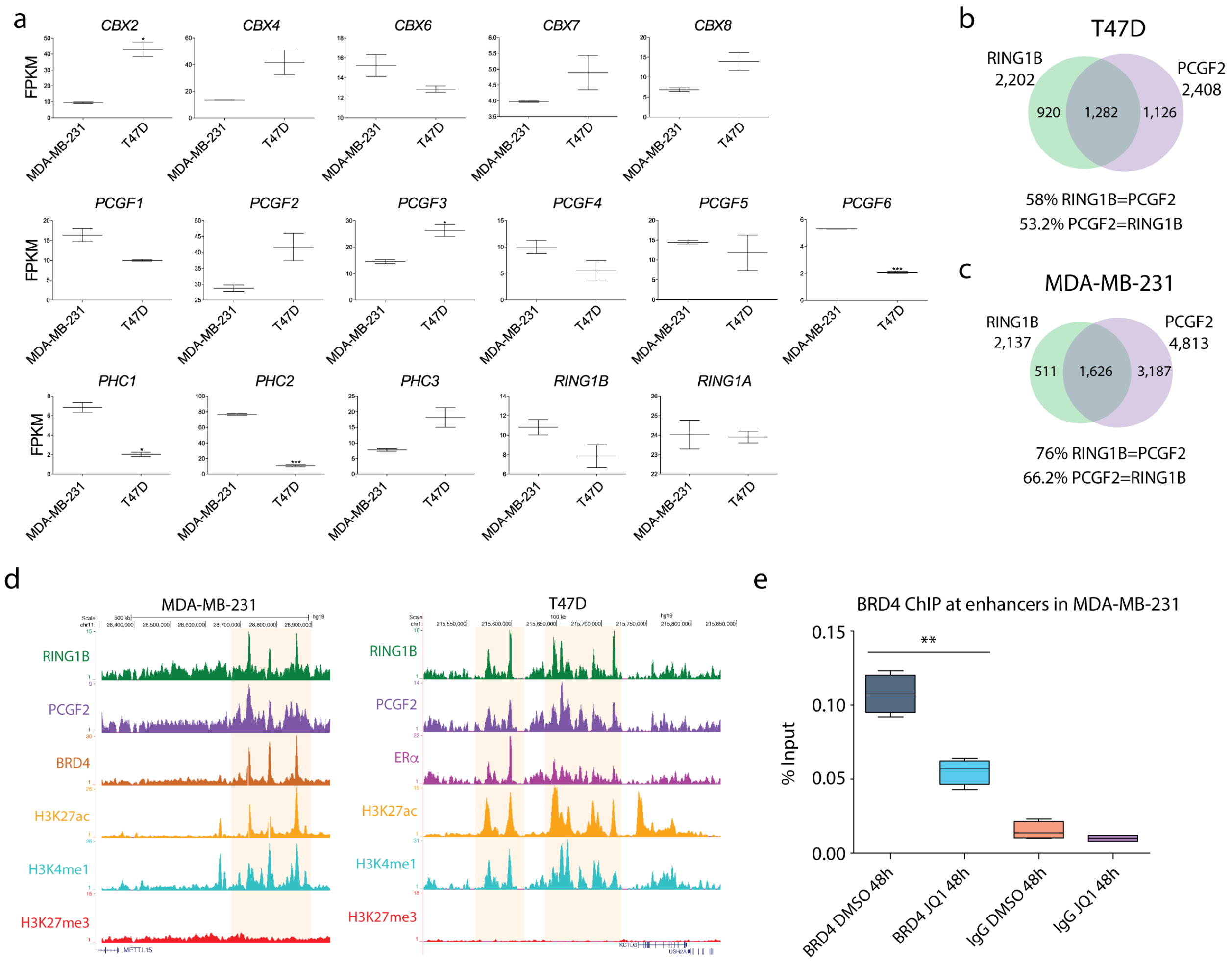

Supplementary Figure 5. PRC1 gene expression and extended analysis of PCGF2 ChIP-seq. (**a**) RNA-seq expression of core PRC1 subunits in T47D and MDA-MB-231 cells represented by FPKM values. (**b**) Venn diagrams of RING1B and PCGF2 target genes in T47D cells. (**c**) Venn diagrams of RING1B and PCGF2 target genes in MDA-MB-231 cells. (**d**) Genome browser screenshots of the profiles of RING1B, ERα, BRD4 and histone modifications at selected SEs. (**e**) BRD4 ChIP-qPCR of BRD4/RING1B-containing enhancers (*BCL2L1, MYC, EGFR* and *GAPDH*) in MDA-MB-231. IgG antibody was used as a negative control. Cells were treated either with vehicle (DMSO) or the BRD4 inhibitor JQ1 for 48hr.

Data is represented as percentage of input immunoprecipitated. Box plots are generated from ChIP signal from four BRD4/RING1B-SEs. Center line of box plots represent the median and upper and lower bounds of whiskers represent the maximum and minimum values, respectively. Significance was determined by two-tailed t-test, ** p-value < 0.01.

Supplementary Figure 6

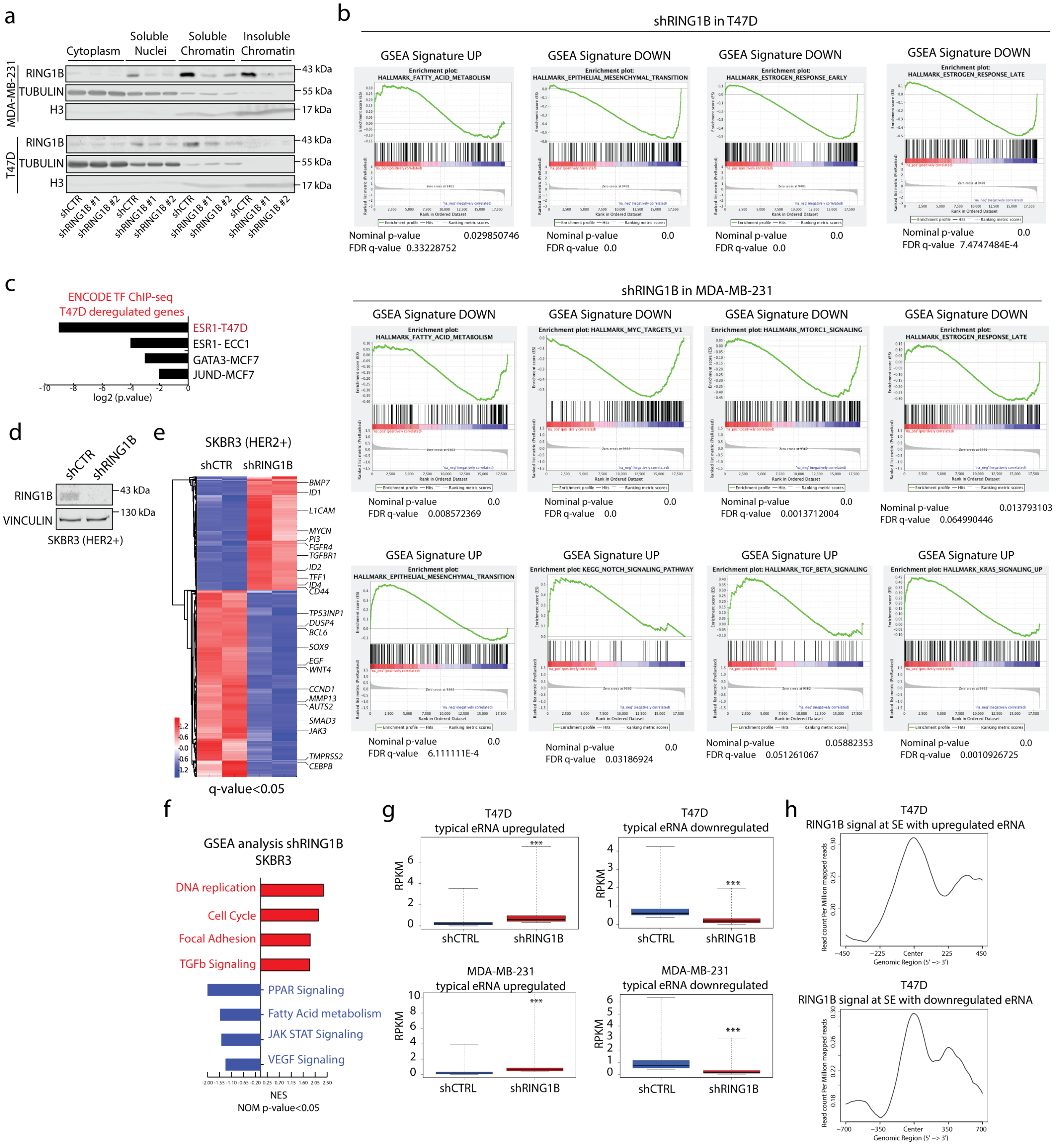

Supplementary Figure 6. Characterization of RING1B depleted T47D, MDA-MB-231 and SKBR3 cells. (**a**) Western blots of fractionated cell extracts from MDA-MB-231 and T47D in control and RING1B knockdown cells. TUBULIN and histone H3 were used as a loading controls. (**b**) Upregulated and downregulated GSEA signature plots in T47D and

MDA-MB-231 cells upon RING1B depletion. (**c**) Enrichr analysis of ENCODE ChIP-seq data sets using only deregulated genes in shRING1B-T47D cells. (**d**) RING1B western blot in control and RING1B-depleted SKBR3 cells. VINCULIN was used as a loading control. (**e**) RNA-seq heatmaps of up- and downregulated genes in RING1B-depleted SKBR3 cells. (**f**) GSEA analyses of shRING1B-SKBR3 cells. RNA-seq experiments were performed from two biological replicates. (**g**) Expression of eRNA in control and RING1B depleted T47D and MDA-MB-231 at typical enhancer regions. Center line of box plots represent the median and upper and lower bounds of whiskers represent the maximum and minimum values, respectively. Significance was determined by Mann-Whitney test, ***, p-value < 0.001. (**h**) RING1B ChIP-seq signal at SE regions with upregulated (top) or downregulated (bottom) eRNA expression in shRING1B-T47D cells.

Supplementary Figure 7

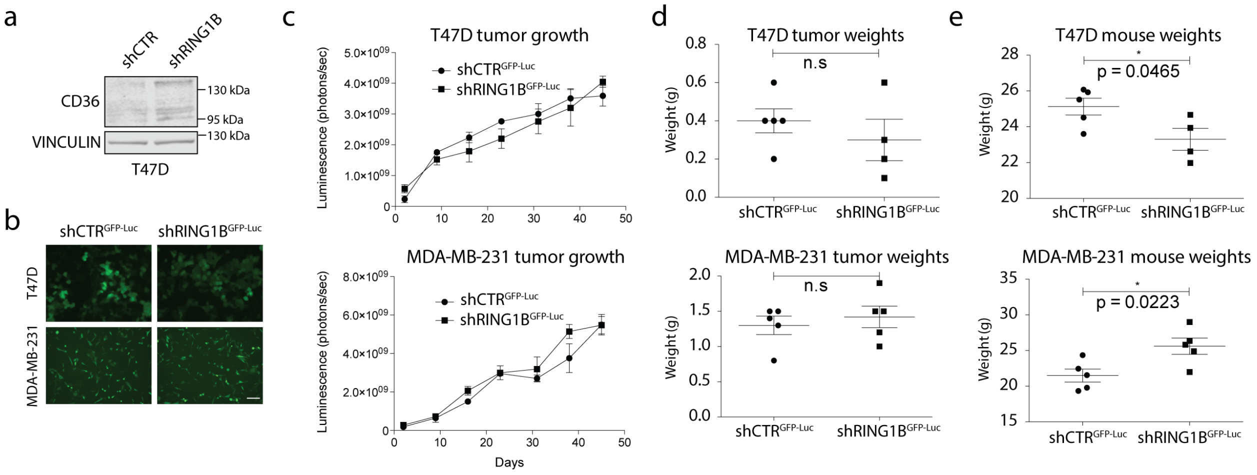

Supplementary Figure 7. Characterization of xenografts experiments. (**a**) CD36 western blot in control and RING1B-depleted T47D cells. VINCULIN was used as a loading control. (**b**) Representative pictures of control and RING1B-depleted T47D and MDA-MB-231 cells expressing GFP-luciferase. Scale bar represents 100µm. (**c**) Quantification of primary tumor growth derived from control and RING1B-depleted T47D and MDA-MB-231 cells expressing GFP-luciferase. Luciferase signal was measured every 7 days by IVIS (n=5/group). (**d**) Final tumor weights derived from control and RING1B-depleted T47D and MDA-MB-231 cells expressing GFP-luciferase. Error bars represent SD and significance was determined by two-tailed t-test. *, p-value < 0.05; n.s., not significant. (**e**) Mice weights at the end of the experiment. Significance was determined by the Mann-Whitney test.

Supplementary Figure 8

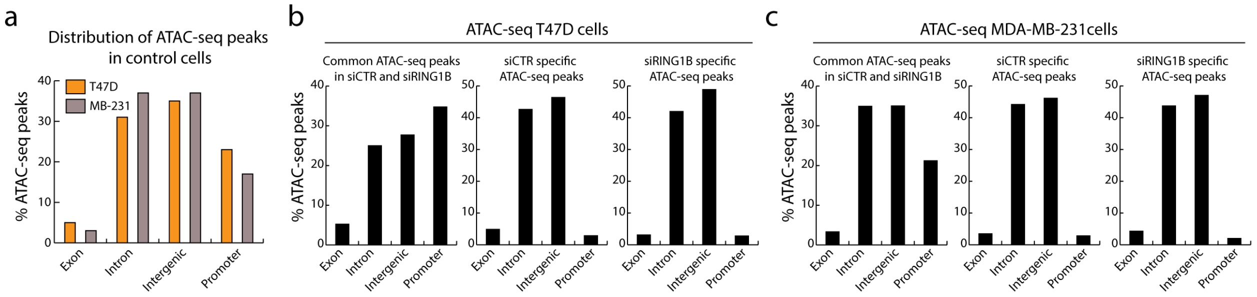

Supplementary Figure 8. Distribution of ATAC-seq peaks. (**a**) Distribution of ATAC-seq peaks in control T47D and MDA-MB-231 cells. (**b, c**) Percentages of common, siCTR-specific, or siRING1B-specific ATAC-seq peaks distributed into exons, introns, intergenic and promoter regions after RING1B depletion in T47D and MDA-MB-231 cells.

Supplementary Figure 9

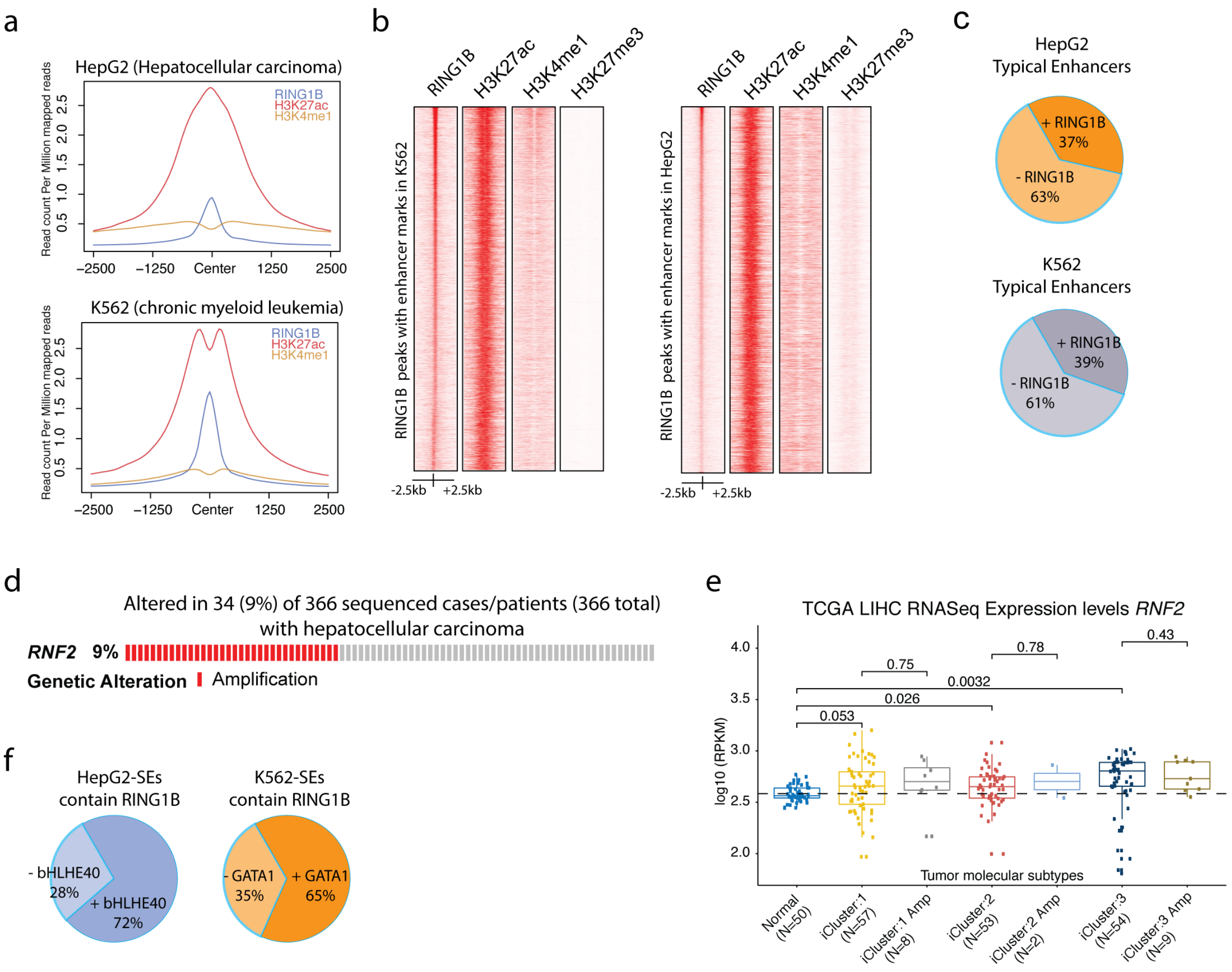

Supplementary Figure 9. RING1B recruitment to enhancers in K562 and HepG2 cells. (**a**) H3K27ac and H3K4me1 ChIP-seq signals relative to the summit (+/– 2.5kb) of RING1B peaks in HepG2 and K562cells. (**b**) Heat maps of RING1B with enhancer histone marks and H3K27me3 in HepG2 and K562 cells. (**c**) Pie charts of typical enhancers with and without RING1B in HepG2 and K562 cells. (**d**) *RNF2* amplification in hepatocellular carcinoma tumors. (**e**) *RNF2* mRNA expression analyzed from ~400 hepatocellular carcinoma tumors extracted from the TCGA liver hepatocellular carcinoma 2017 dataset. Samples were categorized into normal liver tissue and the three molecular hepatocellular carcinoma

subtypes as defined by the TCGA comparing mutational statuses using genome sequencing. Gene expression is represented as log10 (RPKM). The number of patients in each molecular subtype is shown at the bottom. Center line of box plots represent the median and upper and lower bounds of whiskers represent the maximum and minimum values, respectively. Significance was determined using a non-parametric Wilcox test and p-values are indicated above each comparison. (**f**) Pie charts of SEs containing RING1B with and without bHLHE40 in HepG2 cells (left) and RING1B with and without GATA1 in K562 cells.

Supplementary Figure 10

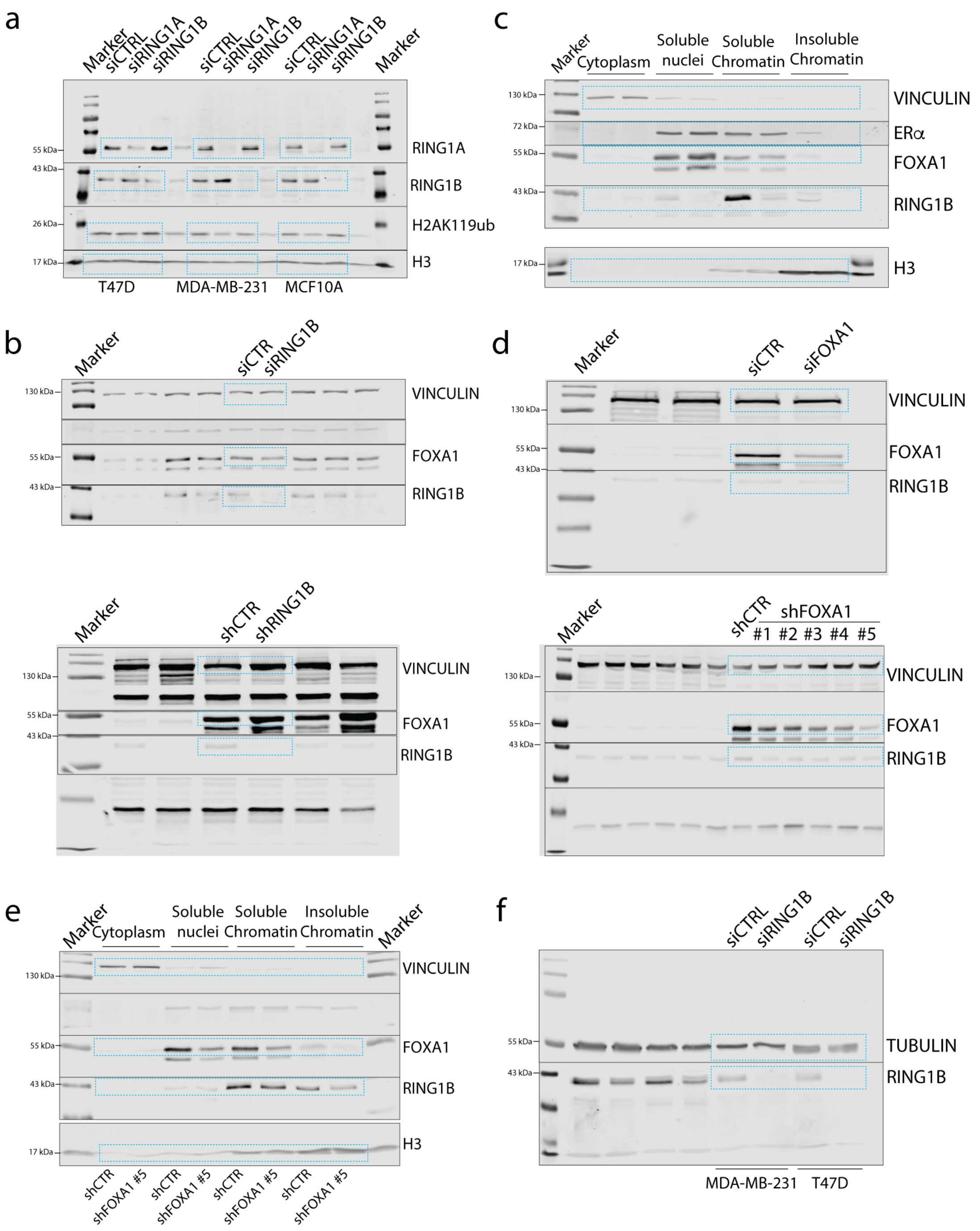

Supplementary Figure 10. Unprocessed blots of main figures. All blots shown are raw image files collected from the Odyssey CLx Imaging System. Solid black lines indicate where membrane was cut prior to incubation with primary antibodies. Dotted blue lines indicate

area of blot used in final figure. Note that exposures may not match final figure to accommodate visualization of all target proteins. Images in final figure contain optimal exposures corresponding to each target protein. All exposures are within the linear range of signal. (**a**) Unprocessed blot corresponding to Fig. 1j. Note the reversed order of the cell lines shown. (**b, top**) Unprocessed blot corresponding to Fig. 5c, left. Note the reversed order of the antibodies. (**b, bottom**) Unprocessed blot corresponding to Fig. 5c, right. Note the reversed order of the antibodies. (**c**) Unprocessed blot corresponding to Fig. 5d. Note that H3 is from a separate western blot of the same samples. (**d, top**) Unprocessed blot corresponding to Fig. 5e, left. (**d, bottom**) Unprocessed blot corresponding to Fig. 5e, right. (**e**) Unprocessed blot corresponding to Fig. 5f. (**f**) Unprocessed blot corresponding to Fig. 6a.
