# Supplementary material for: Polycomb complexes associate with enhancers and promote oncogenic transcriptional programs in cancer through multiple mechanisms
Source: Nat Commun. 2018 Aug 23;9:3377. doi: 10.1038/s41467-018-05728-x (PMC6107513; doi:10.1038/s41467-018-05728-x)
Supplement: Supplementary file 3 — Description of Additional Supplementary Files [file 41467_2018_5728_MOESM3_ESM.pdf]

Description of Additional Supplementary Files

File Name: Supplementary Data 1
Description: RING1B target genes identified in iPSC, MCF10A, MDA-MB-231, and T47D.

File Name: Supplementary Data 2
Description: Genes potentially regulated by super-enhancers identified in MCF10A, MDA-MB-231, and T47D.

File Name: Supplementary Data 3
Description: LC-MS/MS raw data from three independent RING1B and IgG pull-downs in T47D and MDA-MB-231 cells. TR1=T47D replicate 1. MR1=MDA-MB-231 replicate 1.

File Name: Supplementary Data 4
Description: Target genes of ERα in T47D and BRD4 in MDA-MB-231.

File Name: Supplementary Data 5
Description: FPKM values from RNA-seq of MCF10A, MDA-MB-231, and T47D shCTR and shRING1B. rep, replicate.

File Name: Supplementary Data 6
Description: List of antibodies used.

File Name: Supplementary Data 7
Description: Sequences of oligos used for ATAC-seq

File Name: Supplementary Data 8
Description: Sequences of primers used.

File Name: Supplementary Data 9
Description: List of analysis software used.
